# Extracytoplasmic Function Sigma Factors Governing Production of the Primary Siderophores in Pathogenic *Burkholderia* Species

**DOI:** 10.3389/fmicb.2022.851011

**Published:** 2022-02-24

**Authors:** Anne Grove

**Affiliations:** Department of Biological Sciences, Louisiana State University, Baton Rouge, LA, United States

**Keywords:** *Burkholderia*, ECF sigma factor, gene expression, iron acquisition, malleobactin, ornibactin, MftR, siderophore

## Abstract

Bacteria respond to changing environments by modulating their gene expression programs. One of the mechanisms by which this may be accomplished is by substituting the primary σ factor with an alternative σ factor belonging to the family of extracytoplasmic function (ECF) σ factors. ECF σ factors are activated only in presence of specific signals, and they direct the RNA polymerase (RNAP) to transcribe a defined subset of genes. One condition, which may trigger the activation of an ECF σ factor, is iron limitation. To overcome iron starvation, bacteria produce and secrete siderophores, which chelate iron and facilitate its cellular uptake. In the genus *Burkholderia*, which includes several serious human pathogens, uptake of iron is critical for virulence, and expression of biosynthetic gene clusters encoding proteins involved in synthesis and transport of the primary siderophores are under control of an ECF σ factor. This review summarizes mechanisms involved in regulation of these gene clusters, including the role of global transcriptional regulators. Since siderophore-mediated iron acquisition is important for virulence, interference with this process constitutes a viable approach to the treatment of bacterial infections.

## Introduction

Extracytoplasmic function (ECF) σ factors are dedicated to transcription of a subset of bacterial genes in response to environmental change, usually stress (Sineva et al., 2017; Helmann, 2019). By replacing the primary (or “housekeeping”) σ^70^ subunit of the RNA polymerase (RNAP) holoenzyme with an alternative σ factor, the RNAP is directed to transcribe genes whose expression promotes bacterial fitness in response to the perceived signal. The ECF σ factors constitute Group 4 of the σ^70^ family of σ factors. However, while the primary σ^70^ factors feature four domains (σ_1_–σ_4_) for which specific functions have been delineated, the ECF σ factors are more diverse, and they contain only the two domains (σ_2_ and σ_4_) that are required for binding to the core RNAP and to cognate promoters (Feklistov et al., 2014). While this group of proteins was originally named for their involvement in transcription of genes encoding proteins with extracytoplasmic functions (Lonetto et al., 1994), some ECF σ factors have since been described, which control production of proteins with primary functions in the cytoplasm. The number of ECF σ factors encoded within a bacterial genome reflects the likelihood that the bacterium will encounter altered environmental conditions; on average, bacterial genomes encode 6–10 ECF σ factors (Staron et al., 2009; Casas-Pastor et al., 2021).

The classical ECF σ factors are auto-regulatory and co-transcribed with an anti-σ factor, which binds the σ factor directly to sequester it in an inactive state (Staron et al., 2009). The activating signal causes modification or degradation of the anti-σ factor, which results in release of the σ factor to allow its association with the RNAP core enzyme. While anti-σ factors are poorly conserved, many are characterized by a single membrane-spanning region, an N-terminal anti-σ domain located in the cytoplasm, and a variable periplasmic domain (Campbell et al., 2007). Approximately 20% of ECF σ factors appear not to be controlled by an anti-σ factor and to be instead regulated at the level of transcription (Staron et al., 2009).

This review focuses on ECF σ factors, which participate in production of the primary siderophores in pathogenic members of the genus *Burkholderia*. In response to iron starvation, the bacteria produce and secrete siderophores, which facilitate the cellular uptake of iron. Specifically, pathogenic bacteria face a severe iron limitation in a host environment, and siderophores are therefore important for virulence. A picture is emerging, which suggests that several global transcription factors are involved in regulation of these *ecf* genes, possibly to optimize their synthesis in a host environment.

## Siderophores Promote Bacterial Growth in a Host Environment

The transition metal iron is essential due to its involvement as a cofactor or prosthetic group in a number of key proteins such as hemoglobin, cytochromes, and iron–sulfur cluster-containing proteins (Lawen and Lane, 2013). Yet excess iron is toxic due to its chemical reactivity, and improperly sequestered iron can act as a redox catalyst in the production of damaging reactive oxygen species (ROS) through its participation in the Fenton or Haber–Weiss reactions, demanding tight control on iron homeostasis (Abbasi et al., 2021). Fe(II) is soluble in hypoxic, aqueous solution. However, Fe(II) readily gets oxidized to Fe(III), which is poorly soluble, and much of it is stored in the interior cavity of the nanocage ferritin, while circulating Fe(III) is bound to transport proteins such as transferrin and lactoferrin. For bacterial pathogens, this becomes an issue when they colonize the host environment in which iron is sequestered by these high-affinity proteins, a sequestration that maintains free iron at concentrations much below the 10^−8^ to 10^−6^ M, which is required to support the growth of the bacterium (Cassat and Skaar, 2013). In addition, host nutritional immune responses to bacterial infection include efforts to limit iron availability further (Weinberg, 1975).

**Figure 1:**
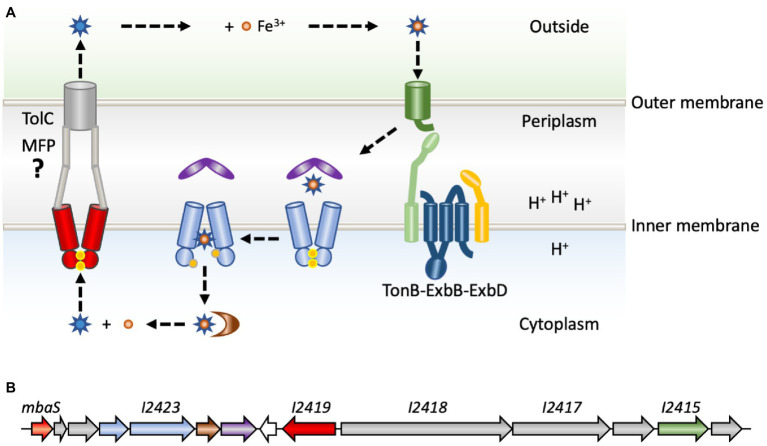
Production and transport of siderophores. **(A)** Newly synthesized and recycled siderophores malleobactin or ornibactin (blue stars) are secreted by a process for which the molecular basis is poorly understood (left). An ATP-binding cassette (ABC) transporter (red) is involved in transport across the cytoplasmic membrane. Transport to the extracellular milieu is speculative and indicated based on comparison to the primary siderophore in pseudomonads, pyoverdine, which is transported by a tripartite system consisting of an ABC permease, a membrane fusion protein (MFP; gray) and an outer membrane transporter, TolC (gray; Hannauer et al., 2010). After the exported siderophore binds Fe^3+^ (orange), the complex is recognized specifically by a membrane-bound TonB-dependent receptor (green). Transport across the outer membrane is fueled by energy furnished by the TonB-ExbB-ExbD complex (light green, blue, and yellow) and the cytoplasmic membrane proton gradient. In the periplasm, the iron–siderophore complex is bound by a periplasmic binding protein (purple), which delivers it to an ABC permease (light blue) for transport across the cytoplasmic membrane, a process that requires ATP hydrolysis (ATP indicated as yellow dots). In the cytoplasm, the siderophore is degraded, or the Fe^3+^ is reduced by ferric iron reductase (brown) to generate Fe^2+^, which has much lower affinity for the siderophore. **(B)** The *mba* gene cluster in *Burkholderia thailandensis*. The gene encoding MbaS (*BTH_I2427*; orange) is predicted to be in an operon with two downstream biosynthetic genes (gray) and the gene encoding the ATP-binding component of the ABC permease (light blue). The gene encoding the ABC permease (*BTH_I2423*; light blue) is predicted to be in an operon with genes encoding the ferric iron reductase (brown) and the periplasmic binding protein (purple). A divergently encoded gene for a hypothetical protein (white) is followed by the gene encoding the cytoplasmic ABC permease (*BTH_I2419*; red). Two genes encoding NRPSs (gray) are in an operon, which also includes a modifying enzyme (gray), a TonB-dependent receptor (*BTH_I2415*; green), and another modifying enzyme (gray); this operon was experimentally verified in *Burkholderia pseudomallei* (Alice et al., 2006).

To counter host nutritional immunity, bacterial pathogens employ a range of mechanisms to optimize the scavenging of iron from the host (Caza and Kronstad, 2013). One of these mechanisms is the production and secretion of secondary metabolites named siderophores, which are small (500–1,500 Da) iron-chelators with affinities for iron that may exceed those of host proteins such as transferrin. Secreted siderophores form high-affinity complexes with Fe(III), and the complexes are then transported back into the bacterial cytoplasm by specific transporters (Crosa and Walsh, 2002; Hider and Kong, 2010). This transport process involves TonB-dependent outer membrane receptors recognizing the iron-siderophore complexes specifically and with high affinity, following which the iron-chelate enters the periplasm where it is bound by a periplasmic binding protein. The metal complexes are subsequently transported across the cytoplasmic membrane by ATP-binding cassette (ABC) permeases (Figure 1A; Caza and Kronstad, 2013). TonB-dependent receptors are so named because they make contact with the TonB component of the TonB-ExbB-ExbD complex (Braun and Endriss, 2007; Celia et al., 2019). TonB-ExbB-ExbD is a molecular motor, which utilizes the proton motive force across the cytoplasmic membrane of Gram-negative bacteria to furnish energy to the outer membrane transporters, thereby facilitating uptake of required nutrients, such as iron-siderophore complexes. Once in the cytoplasm, the iron must be released, which may involve hydrolysis of the siderophore by esterases to liberate Fe(III). Alternatively, Fe(III) may be reduced to Fe(II) by ferric reductases, as ferrous iron has much lower affinity for the siderophore (Trindade et al., 2021).

Pathogens typically produce multiple siderophores, which may have redundant functions or play specific roles depending on environment or the route of infection (Takase et al., 2000). Some are produced by the modular non-ribosomal peptide synthetases (NRPS), whereas others are the product of NRPS-independent siderophore synthases (Crosa and Walsh, 2002; Carroll and Moore, 2018). Biosynthetic gene clusters encoding proteins responsible for production and transport of secondary metabolites such as siderophores are often large, and the genes are subject to tight regulation (Thapa and Grove, 2019). Such regulation may involve global transcriptional regulators, which generally regulate a large number of genes with distinct functionalities, and/or it may depend on local cluster-specific regulators, which are dedicated to a certain pathway.

## Siderophores Produced by Pathogenic *Burkholderia* Species

The genus *Burkholderia* includes a number of serious human pathogens. Phylogenetic analyses have suggested the division of *Burkholderia* species into distinct clades, one of which comprises plant and human pathogens (Sawana et al., 2014). Within this lineage, members of the *Burkholderia cepacia* complex (Bcc) include plant pathogens as well as species, which have emerged as dangerous opportunistic pathogens, such as *Burkholderia cenocepacia*, which can cause life-threatening infections in the lungs of persons living with cystic fibrosis or chronic granulomatous disease (Scoffone et al., 2017). The *Burkholderia pseudomallei* complex (Bpc) pathogens include *B. pseudomallei* and its clonal derivative *Burkholderia mallei*, both of which infect both humans and animals, causing melioidosis in humans and glanders in animals, respectively (Tapia et al., 2019). *Burkholderia pseudomallei* is a soil saprophyte, while *B. mallei* is an obligate pathogen. Because both species are highly contagious, inherently resistant to many antibiotics, and associated with a high mortality rate, they are classified as Tier 1 Select Agents. Therefore, many virulence traits are analyzed in the Bpc species *Burkholderia thailandensis*, which is much less virulent despite sharing significant genetic similarity with the more pathogenic species (Haraga et al., 2008).

All pathogenic *Burkholderia* species must acquire iron from their host, and they produce several siderophores (Butt and Thomas, 2017). The primary siderophore in Bcc species is thought to be the linear hydroxamate/hydroxycarboxylate ornibactin, which bears some similarity to the pyoverdines produced by pseudomonads, while Bpc species produce the structurally similar malleobactins. Accordingly, the biosynthetic gene clusters are also similar, and disruption of genes within these clusters is associated with reduced virulence (Sokol et al., 2000; Visser et al., 2004; Wand et al., 2011). In addition, secondary siderophores such as pyochelin are produced in many species, and these compounds are thought to have lower affinity for iron compared to ornibactin and malleobactin. The secondary siderophores may be more important in environmental conditions and not in virulence, as exemplified by the genomic deletion of the pyochelin biosynthetic gene cluster in the obligate pathogen *B. mallei* (Esmaeel et al., 2016).

The biosynthetic gene clusters involved in *B. cenocepacia* ornibactin and *B. pseudomallei* malleobactin production have been characterized in detail, and homologous gene clusters have been analyzed in *Burkholderia xenovorans* and *B. thailandensis* (Figure 1B; reflecting the annotated *mba* biosynthetic gene cluster in *B. thailandensis*; Agnoli et al., 2006; Alice et al., 2006; Franke et al., 2013; Vargas-Straube et al., 2016). These gene clusters are conserved among species and are usually encoded on the larger chromosome. Genes contained within these clusters encode two NRPSs as well as several modifying enzymes (Figure 1B; gray), an ABC transporter implicated in export of siderophores (red), and a set of proteins dedicated to uptake of iron-siderophore complexes. The latter comprise a TonB-dependent outer membrane receptor (green), a periplasmic binding protein (purple), and a cytoplasmic ABC transporter (light blue). The first gene in the clusters encodes an ECF σ factor referred to as MbaS (for malleobactin sigma) in *B. pseudomallei* and OrbS (for ornibactin sigma) in *B. cenocepacia* (Agnoli et al., 2006; Alice et al., 2006). Notably, none of the adjacent genes are predicted to encode an anti-σ factor.

## ECF σ Factors Dedicated to Production of Ornibactin and Malleobactin

MbaS and OrbS belong to a specific subgroup of ECF σ factors. Phylogenetic analyses have been used to classify all bacterial ECF proteins. They were first organized into 43 distinct groups for which specific functions were noted, and this classification was subsequently expanded to comprise 157 phylogenetic groups (Staron et al., 2009; Casas-Pastor et al., 2021). Based on these classifications, ECF σ factors belonging to the originally proposed groups ECF05-ECF09 (merged to generate ECF243 according to the updated classification) are generally involved in iron acquisition. It should be noted that the description of the more recently defined group ECF243 suggests that the genomic context includes an anti-σ factor, which does not accurately reflect the gene clusters under control of OrbS and MbaS; these σ factors were part of the originally described ECF09 family for which the apparent absence of a cognate anti-σ factor was a defining feature. Instead, genes encoding ECF09-family proteins are often adjacent to genes ending MbtH-like proteins (Figure 1B), which generally associate with the adenylation domain of NRPS enzymes (Felnagle et al., 2010).

OrbS was originally identified based on its 40% sequence identity to PvdS (for pyoverdine sigma), which controls biosynthetic genes involved in pyoverdine production in pseudomonads (Cunliffe et al., 1995; Llamas et al., 2014). PvdS has been extensively analyzed in species such as the opportunistic pathogen *Pseudomonas aeruginosa*, where it controls not only production and transport of pyoverdine but also production of virulence factors such as exotoxin A. Expression of the PvdS regulon requires pyoverdine, which signals to the anti-σ factor FpvR, resulting in activation of PvdS (Lamont et al., 2002; Llamas et al., 2014). Despite sequence similarities, regulation of OrbS and MbaS is clearly different from that of PvdS with no evidence for the involvement of an anti-σ factor or the respective siderophores; instead, production of these ECF σ factors appears to rely on transcriptional regulation of the respective genes. Compared to PvdS, OrbS has a 29-amino acid N-terminal extension, and it appears to control only genes within the ornibactin biosynthetic gene cluster (Agnoli et al., 2019).

### Regulation of *orbS* and *mbaS* by Fur

**Figure 2:**
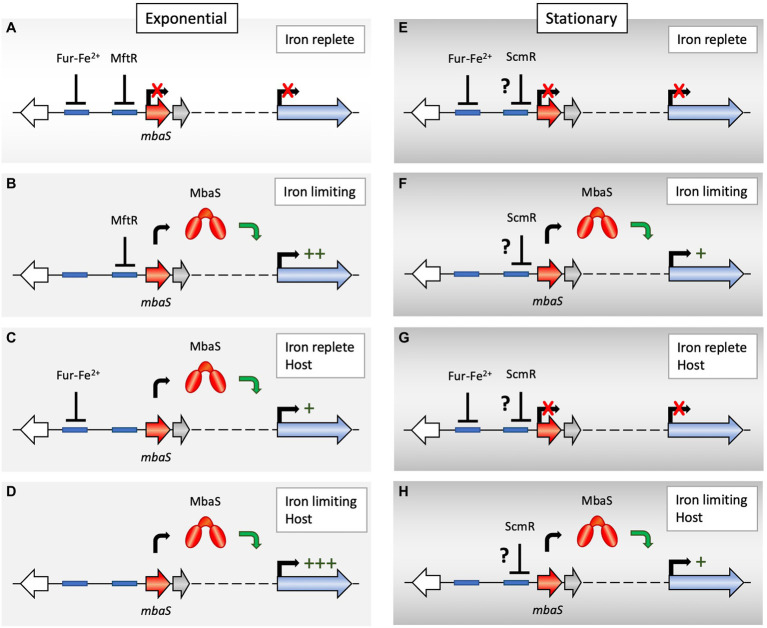
Proposed regulation of *mbaS* expression. **(A)** During exponential growth under iron-replete, environmental conditions, *mbaS* (which is predicted to be in an operon with three downstream genes; dotted line) is repressed by Fe^2+^-bound ferric uptake regulator (Fur) and by major facilitator transport regulator (MftR). As a consequence, MbaS-dependent genes within the *mba* gene cluster (blue arrow) are not expressed, and no malleobactin is produced. Blue boxes represent predicted binding sites for Fur and MftR. **(B)** During exponential growth in an iron-limited environment, the Fur-Fe^2+^ complex dissociates, and Fur leaves the *mbaS* promoter, which is subject to repression only by MftR. The attenuated repression allows production of MbaS, which directs transcription of MbaS-dependent genes within the *mba* gene cluster, leading to malleobactin production and transport. **(C)** In an iron-replete host environment, MftR binds its ligands xanthine or urate, which are produced during the oxidative burst generated by host cells, and MftR abandons the *mbaS* promoter. Fur-Fe^2+^ maintains *mbaS* expression at a low level. **(D)** In an iron-limited host environment, characterized by the presence of elevated levels of xanthine and urate, dissociation of both Fur and MftR leads to maximal *mbaS* expression. **(E,F)** During stationary phase, regulation by Fur is also iron-dependent. Because xanthine accumulates due to activation of purine salvage, MftR binds xanthine and is unable to bind DNA. Instead, ScmR may bind the *mbaS* promoter to reduce MbaS production, in turn limiting siderophore production during high cell density. Binding of ScmR to the *mbaS* promoter only in stationary phase may be due to higher cellular levels of ScmR in stationary phase or due to mutually exclusive binding of ScmR and MftR. Direct binding of ScmR is speculative as a cognate site cannot be predicted, and it is conceivable that a separate transcriptional regulator under control of ScmR instead binds the *mbaS* promoter (as indicated by the question mark). **(G,H)** ScmR is likewise predicted to attenuate siderophore production in a host environment under conditions of high cell density.

Genes encoding proteins involved in iron acquisition are generally expressed only when iron is limiting. When iron is present, Fe(II) reversibly binds the global transcriptional regulator ferric uptake regulator (Fur), which facilitates the binding of Fur to its cognate sites (the Fur-box), repressing genes involved in iron uptake (Baichoo and Helmann, 2002; Fillat, 2014). In keeping with this theme, transcription of the *orbS* gene was shown to be repressed by Fur, and a sequence with homology to the consensus Fur-box was identified within the σ^70^-dependent *orbS* promoter (Agnoli et al., 2006). Fur was not seen to bind the OrbS-dependent promoters within the ornibactin biosynthetic gene cluster, indicating that increased ornibactin production in response to iron limitation depends on Fur-mediated regulation of *orbS*. Similarly, transcription of genes within the *B. pseudomallei* malleobactin biosynthetic gene cluster requires MbaS, and the *mbaS* promoter contains a Fur-box, which is recognized by *Escherichia coli* Fur (Alice et al., 2006). A Fur-box is also seen in the *B. thailandensis mbaS* promoter, centered 55 bp upstream of the annotated start codon (Figure 2A). In *B. xenovorans*, a putative Fur-box was also detected within the promoter driving expression of the ECF σ factor (Vargas-Straube et al., 2016). Thus, production of OrbS and MbaS, and hence expression of the corresponding biosynthetic gene clusters, is favored under conditions of iron limitation. The homologous *pvdS* from pseudomonads is likewise controlled by Fur in an iron-dependent manner (Leoni et al., 1996).

### Regulation by the Global Transcriptional Regulators ScmR and MftR

In *B. thailandensis*, two global transcriptional regulators have recently been implicated in regulating expression of biosynthetic gene clusters involved in production of secondary metabolites. Inactivation of the gene encoding the secondary metabolite regulator ScmR, a LysR-type transcriptional regulator (LTTR), resulted in differential expression of a number of biosynthetic gene clusters, including the malleobactin (*mba*) gene cluster; notably, the *mba* genes were upregulated ~4-fold in stationary phase in *ΔscmR* cells, indicating that ScmR directly or indirectly functions as a repressor, but not during exponential growth (Mao et al., 2017; Le Guillouzer et al., 2020). Expression of *scmR* is stimulated by quorum sensing, which might result in increased production of ScmR and hence more efficient repression of the malleobactin biosynthetic gene cluster in stationary phase, offering one possible explanation for why deletion of *scmR* only causes increased gene expression in stationary phase. It should be noted that this interpretation implies a relatively low-affinity binding of ScmR to its cognate site, necessitating higher cellular levels of ScmR for efficient binding. Consistent with this interpretation, the *B. thailandensis* malleobactin biosynthetic gene cluster was previously reported to be repressed by quorum sensing during stationary phase, and ornibactin production in *B. cepacia* was shown to increase on inactivation of quorum sensing (Lewenza and Sokol, 2001; Majerczyk et al., 2014). With a consensus motif of T-N_11_-A for DNA binding by LTTRs (Maddocks and Oyston, 2008), it is not possible to predict if ScmR is likely to bind the *mbaS* promoter directly.

The other global transcriptional regulator shown to control production of secondary metabolites is major facilitator transport regulator (MftR), which is a member of the multiple antibiotic resistance regulator (MarR) protein family (Gupta et al., 2019). MftR is conserved among pathogenic Bpc and Bcc species, but not in environmental species such as *B. xenovorans* (Gupta et al., 2019). Inactivation of *B. thailandensis mftR* also results in differential expression of numerous biosynthetic gene clusters (Gupta et al., 2017). Specifically, *mbaS* expression is increased ~5-fold in *ΔmftR* cells during exponential growth, indicating that MftR functions as a repressor. Consistent with increased expression of the malleobactin gene cluster, *ΔmftR* cells produce increased levels of siderophores. MftR binds an 18 bp palindromic sequence (Gupta and Grove, 2014), and a sequence with similarity to this palindrome is centered 24 bp upstream of the *mbaS* start codon and downstream of the predicted Fur-box (Figure 2A). An interpretation, which is consistent with available data is therefore that both Fur and MftR simultaneously bind the *mbaS* promoter to repress transcription during exponential phase and in iron-replete media, conditions under which ScmR does not appear to affect *mbaS* expression. MftR also represses expression of *scmR*, as indicated by an ~3-fold increased expression in *ΔmftR* cells, whereas inactivation of *scmR* has no effect on *mftR* expression (Gupta et al., 2017; Mao et al., 2017). Cellular levels of ScmR may therefore be elevated in *ΔmftR* cells, possibly attenuating *mbaS* expression in this strain.

According to this model for regulation of *mbaS* expression, maximal expression would be predicted to occur when both Fur and MftR dissociate. Under conditions of iron limitation, the Fur-Fe^2+^ complex dissociates, and Fur would leave the *mbaS* promoter, resulting in increased *mbaS* expression, with MbaS subsequently directing RNAP to MbaS-dependent promoters within the *mba* gene cluster (Figure 2B). MftR binds DNA in absence of ligands; however, the purine catabolites xanthine and urate bind directly to MftR, resulting in attenuated DNA binding and upregulation of target genes (Gupta and Grove, 2014; Gupta et al., 2017; Thapa and Grove, 2021). These purines are relevant in a host environment (Figure 2C); when bacteria infect a host, a non-specific oxidative burst occurs in which ROS are produced by enzymes such a NADPH oxidase and xanthine dehydrogenase (Xdh). The latter participates in normal purine metabolism, converting hypoxanthine to xanthine and xanthine to urate. During infection, this enzyme is modified, resulting in its transfer of electrons to molecular oxygen and the production of ROS. Accordingly, both xanthine and urate accumulate during the infection process (Segal et al., 2000; Nishino et al., 2008; Crane et al., 2013; Ma et al., 2016). The dual regulation of *mbaS* by Fur and MftR may therefore exist to ensure that *mbaS* expression is most effectively upregulated in an iron-limited host environment (Figure 2D).

In stationary phase, *mbaS* expression is repressed by ScmR (Figures 2E–H; Mao et al., 2017). As noted above, this could be explained by increased cellular levels of ScmR leading to more efficient binding, either to the *mbaS* promoter or to the promoter of another regulatory protein. However, it is also conceivable that ScmR binds directly to the *mbaS* promoter, and that MftR and ScmR binding is mutually exclusive. According to this scenario, MftR binding during exponential growth might preclude ScmR binding, explaining why inactivation of *scmR* has no effect on *mbaS* expression. In stationary phase, a stringent response will be elicited, one consequence of which is a reliance on purine salvage over *de novo* biosynthesis. This has been shown to include an upregulation of the operon encoding Xdh, which is important for purine salvage (Hillerich and Westpheling, 2008; Sivapragasam and Grove, 2016). In *B. thailandensis*, upregulation of *xdh* correlates with increased cellular levels of xanthine and with upregulation of specific MftR target genes (Thapa and Grove, 2021). Dissociation of xanthine-bound MftR during stationary phase might therefore in turn permit binding of ScmR to *mbaS* promoters abandoned by MftR (Figures 2E–H). Regardless of the mechanism by which ScmR controls *mbaS* in stationary phase only, its purpose may be to prevent excessive accumulation of siderophores.

## Conclusion

Production of malleobactin and ornibactin requires expression of large biosynthetic gene clusters, which demands significant cellular resources. Accordingly, tight transcriptional control is exerted. These siderophores have high affinity for Fe^3+^ and may be of particular importance in the iron-limited host environment. A picture is emerging suggesting that regulation of genes encoding MbaS and OrbS determines availability of these ECF σ factors and not anti-σ factors or the respective siderophores. Notably, the involvement of the global regulator MftR, which has been implicated in controlling genes associated with virulence, suggests that regulatory mechanisms are optimized to ensure maximal expression when two criteria are met—iron limitation and host colonization (Figure 2). However, maximal does not equal optimal. The quorum sensing-dependent repression by ScmR during stationary phase appears to function as an overflow valve, precluding excessive production of high-affinity iron chelators under conditions of high cell density.

## Author Contributions

AG contributed to conceptualization, writing—original draft, writing—review and editing, visualization, and funding acquisition.

## Funding

This work was supported by the National Science Foundation (MCB-1714219 to AG).

## Conflict of Interest

The author declares that the research was conducted in the absence of any commercial or financial relationships that could be construed as a potential conflict of interest.

## Publisher’s Note

All claims expressed in this article are solely those of the authors and do not necessarily represent those of their affiliated organizations, or those of the publisher, the editors and the reviewers. Any product that may be evaluated in this article, or claim that may be made by its manufacturer, is not guaranteed or endorsed by the publisher.
